# Associations of environmental tobacco smoke with ADHD and executive function in early adulthood: results from a cross-sectional study

**DOI:** 10.3389/fpsyt.2025.1733240

**Published:** 2026-01-14

**Authors:** Yunyun Liu, Hong Ge, Yanling Shu, Mingyang Wu

**Affiliations:** 1 Department of Preventive Medicine, School of Medical Technology, Jiangsu College of Nursing, Huai’an, Jiangsu, China; 2 Department of Maternal and Child Health, School of Public Health, Tongji Medical College, Huazhong University of Science and Technology, Wuhan, China; 3 Department of Maternal and Child Health, Xiangya School of Public Health, Central South University, Changsha, China

**Keywords:** attention-deficit/hyperactivity disorder, early adulthood, executive function, secondhand smoke, thirdhand smoke

## Abstract

**Background:**

Secondhand smoke (SHS) and thirdhand smoke (THS) have been shown to increase the risk of physical health and mental health. However, the impact of SHS and THS exposure on ADHD symptoms and executive function remain to be elucidated.

**Methods:**

Online surveys were used to recruit participants at a Chinese vocational college. The frequency of contact with smokers or detecting tobacco odors in residential environments was used to define SHS exposure, whereas the frequency of contact with surfaces contaminated by smokers was used to define THS exposure. The associations of SHS and THS exposure with the risk of ADHD symptoms and the executive function (Behavioral Regulation Index, BRI; Metacognitive Index, MI; Global Executive Composite, GEC) scores were evaluated by logistic regression analysis or linear regression analysis.

**Results:**

The rate of SHS and THS exposure was 87.7% and 76.9%, with 8.2% stating SHS exposure ≥15 min at least 1 day/week. After adjustment for confounding variables, SHS exposure (≥15 minutes on ≥1 day/week) had higher odds of ADHD symptoms (OR, 1.31; 95% CI, 1.02–1.67), and higher BRI score (β, 2.34; 95% CI, 0.95–3.73), higher MI score (β, 3.12; 95% CI, 1.29–4.95), higher GEC score (β, 5.46; 95% CI, 2.26–8.66). Additionally, the higher the frequency of SHS/THS exposure and the greater the number of surrounding smoker, the higher risk of ADHD symptoms and the poorer executive function (*P* trend <0.001).

**Conclusions:**

SHS and THS exposure were related to the occurrence of ADHD symptoms and impaired executive function among young adults at a Chinese vocational college in this cross-sectional study. Further researches are warranted to validate these associations in more diverse populations.

## 1 Introduction

Global data indicate that nearly one in three non-smoking young adults is regularly exposed to environmental tobacco smoke (ETS) in homes, workplaces, and social venues (1). The main components of ETS are secondhand smoke (SHS) and thirdhand smoke (THS), which seriously impact public health in nonsmokers worldwide (2). In Asian country, the high prevalence of SHS in public places was also reported (3). For example, the SHS exposure rate in the workplaces and the household was 50.9% and 44.9% in China in 2018 (4), and the rate of THS exposure are 47.8% among young people in China (5). SHS is composed of the smoke emitted from burning tobacco products (sidestream smoke) and exhaled by smokers (mainstream smoke) (2). THS, consists of residual tobacco smoke contaminants that adsorb to surfaces, dust, and fabrics, persisting for months or even years after active smoking has ceased. These contaminants can re-emit volatile compounds or be resuspended into the air, creating secondary pollution (6). SHS and THS have been shown to increase the risk of different health effect in nonsmokers. A systematic review summarized the disease burden caused by SHS exposure, including but not limited to cardiovascular disease, cancer, respiratory conditions, type 2 diabetes (7). Similarly, the dangers of THS have been reported in recent years. Evidence showed that THS not only lead to DNA damage but also increase cancer incidence rate in non-smokers and causes cytotoxicity in mouse (8, 9). Recently, emerging evidence suggests that the health impact of SHS and THS may extend to neurological and cognitive domains, particularly in vulnerable populations such as children and adolescents (10, 11).

Attention-deficit/hyperactivity disorder (ADHD) is a neurodevelopmental disorder characterized by persistent patterns of inattention, hyperactivity, and impulsivity, which is no longer regarded as a childhood-limited condition, 50–65% of children diagnosed with ADHD continue to manifest clinically significant symptoms into early adulthood (12). Beyond the hallmark symptoms, young adults diagnosed with ADHD frequently exhibit pronounced deficits in executive function (13). Executive function is a high-order cognitive processes responsible for goal-directed and self-regulation behaviors that encompasses three core domains: working memory, inhibitory control, and cognitive flexibility (14), which are critical for navigating the demands of early adulthood, including academic achievement, career development, and social relationships.

A growing body of research have linked SHS exposure during prenatal development and childhood to an increased risk of ADHD symptoms and executive function impairments in pediatric populations (15–21). For example, early-life exposure to SHS is associated with reduced inhibitory control in children (20). Similarly, childhood exposure to SHS had negative impact on behavioral problems over time (21). The potential carcinogenic risk posed by THS exposure have been reported in human and animal study (22, 23). However, the evidence of potential impact of SHS and THS on ADHD and executive function in early adulthood is limited and the underlying mechanism remains understudied. The toxicants released by SHS and THS have been shown to disrupt the development of prefrontal cortex circuits, which are critical for the maturation of executive function (24). Therefore, we hypothesize that SHS and THS exposure may contribute to an increased risk of ADHD symptoms primarily through its detrimental effect on the development of executive functions.

In this study, we aimed to explore the associations of SHS and THS exposure with ADHD and executive function among college student, which was selected as the target population because of the transition into independent living, higher education, and the workforce, and relatively high prevalence of mental health (25). Any cognitive impairment during this stage can have far-reaching consequences for their long-term well-being and life outcomes thus elucidating these associations could enhance our understanding of the neurocognitive risks of SHS/THS exposure among young adults and inform targeted public health interventions.

## 2 Methods

### 2.1 Study population

Between October and November 2024, a cross-sectional survey was administered to recruit participants at a vocational college in Jiangsu, China, and the first follow-up data collection was completed from April to May 2025. In the baseline data, we collected demographic information and SHS/THS exposure of the participants. In the follow-up data, we collected the symptoms of ADHD and the executive function of the participants. All 144 classes were scheduled to participate in this study. Students completed the questionnaire by scanning a unique Quick Response Code (QR Code). The questionnaire was programmed with forced-response checks, preventing submission until critical variables was completed to ensure no missing data. After excluding participants who refused to participate, 6474 individuals ultimately completed the full electronic questionnaire. Each subject provided informed consent, and all procedures of this study adhered to the Declaration of Helsinki. According to the exclusion criteria: (1) participants whose demographic information were duplicated or missing or health-examination data were internally inconsistent (N = 558), (2) participants who answered quality control questions incorrectly (e.g., numerical calculation questions and similar judgment questions) (N = 157), (3) participants who were active smokers or former smokers (N = 43), (4) participants who were lost during the first follow-up (N = 1703), a total of 4013 participants were included in this study finally. The study protocol was granted by the Ethics Committee of Jiangsu College of Nursing (JSCN-ME-2024040202).

### 2.2 Assessments of exposure

Exposure of SHS/THS was assessed referring to the Global Adult Tobacco Survey and prior investigations in China, and suitable revisions were made to fit our study. Internal consistency of the SHS/THS exposure assessment was acceptable (Cronbach’s α = 0.72). The duration of SHS exposure was assessed using question: Have you ever experienced smoke exhaled by others (≥15 minutes on ≥1 day/week), which was classified as yes or no. The frequency of SHS/THS exposure was assessed using question: Have you ever seen other people smoking or experienced tobacco smells in your living/working environment, which was classified as six groups: very frequent (≥1/day), fairly frequent (5–6/week), usually (3–4/week), infrequent (1–2/week), very infrequent (1–3/month), and never. The number of surrounding smokers was assessed using question: How many smokers are present in your living/working environment, which was classified as four groups: 0, 1–4, 5–9, and ≥10.

### 2.3 Assessments of outcome

The symptoms of ADHD were assessed by the Adult ADHD Self-Report Scale (ASRS) which showed the highest sensitivity (0.9) and specificity SP (0.88) among all ADHD rating scales (26). The completed version of ASRS consists of 18 questions about frequency of recent DSM-IV Criterion A symptoms of adult ADHD, which was divided into Part A (focusing on attention deficit symptoms, 9 questions) and Part B (focusing on hyperactivity/impulsivity symptoms, 9 questions). Each question asked how often a symptom occurred over the past 6 months on a 0–4 scale with responses of never (0), rarely (1), sometimes (2), often (3), and very often (4), and total score equal to or of higher than 17 is defined as possible ADHD symptoms (26).

The Behavior Rating Inventory of Executive Function–Adult Version (BRIEF-A) was used to measure the executive function of the participants. The BRIEF–A is an effective measurement that assesses nine domains of executive function. The difficulties with daily activities are recorded by participants, yielding a total score Global Executive Composite (GEC) as well as two composite indices scores, the Behavioral Regulation Index (BRI) and the Metacognitive Index (MI) (27). The BRI comprises four scales (inhibit, shift, emotional control and self-monitor), and the MI comprises five scales (initiate, working memory, plan/organize, task monitor and organization of materials). Participants answered 75 items using a 3-point Likert scale (1 = never, 2 = sometimes, 3 = often) to show the frequency of each question becoming a problem in the past month. The higher the score, the degree of executive dysfunction (27).

### 2.4 Covariates

The demographic and behavioral covariates were selected according to previous research and our preliminary data analysis using stepwise regression: age, sex, race, BMI, parental education, home address, physical activity, academic stress, and alcohol consumption (28–32). Parental education was categorized into three groups: junior high school or less, high school, college or above. Household income was classified as four groups: < 30,000 yuan, 30,000 to 80,000 yuan, 80,000 to 150,000 yuan, ≥150,000 yuan. Physical activity was measured by self-reported participation in energetic leisure activities in a week. Academic stress was classified as seven groups: none, low, somewhat low, moderate, somewhat high, high, or unknown/prefer not to answer. BMI was computed as body weight (kg) divided by height squared (m^2^).

### 2.5 Statistical analysis

Continuous variables with normal distributions were expressed as mean (standard deviation, SD), whereas categorical variables were presented as number and percentages. Logistic regression model was used to estimate the odds ratios (ORs) and 95% confidence intervals (CIs) to assess the associations of SHS and THS exposure with ADHD symptoms. Linear regression model was applied to assess the associations of SHS and THS exposure with BRIEF-A scores. We checked normality, homoscedasticity, multicollinearity in these regression models. Trend test was conducted to test dose-response relationship between SHS/THS exposure and ADHD symptoms and executive function. Covariates for adjustment in the logistic regression model and linear regression model were selected according to the previous studies and stepwise selection, including age, sex, race, BMI, parental education, home address, physical activity, academic stress, and alcohol consumption. All statistical analyses were performed with R software (version 4.5.0). A two-tailed *P* value < 0.05 was considered statistically significant.

## 3 Results

### 3.1 Participant characteristics

The main characteristics of the study participants were summarized in **Table 1**. The mean age of study participants was 19.2 years. Of the 4013 study participants, although only 8.2% reported SHS exposure ≥15 min at least 1 day/week, 87.7% and 76.9% reported SHS and THS exposure according to the frequency measurements. The occurrence of ADHD symptoms was 24.4%. The mean (SD) of BRIEF-A scores was 11.5 (12.5) for BRI, 15.9 (16.5) for MI, 27.4 (28.9) for GEC. Compared with participants without ADHD symptoms, participants with ADHD symptoms were more likely to experience higher academic stress, physical inactivity, alcohol consumption, and lower parental educational levels. Higher scores of BRIEF–A scales (BRI, MI and GEC) was found in participants with ADHD symptoms. The mean (SD) of BRI, MI, and GEC scores in participants with ADHD symptoms were 25.0 (10.3), 33.4 (13.5), and 58.4 (23.7).

**Table 1:** The characteristics of the study population.

Characteristics	Total	ADHD symptoms		*P* value
		No	Yes	
Age, mean (SD), y	19.2 (0.7)	19.2 (0.7)	19.2 (0.7)	0.703
BMl, mean (SD), kg/m²	22.3 (4.2)	22.3 (4.2)	22.4 (4.3)	0.701
Sex, n (%)				0.006
Male	517 (12.9)	366 (70.8)	151 (29.2)	
Female	3496 (87.1)	2668 (76.3)	828 (23.7)	
Race, n (%)				0.725
Han nationality	3938 (98.1)	2976 (75.6)	962 (24.4)	
Other	75 (1.9)	58 (77.3)	17 (22.7)	
Home address				0.437
Urban	1596 (39.8)	1217 (76.3)	379 (23.7)	
Rural	2417 (60.2)	1817 (75.2)	600 (24.8)	
Paternal educational, n (%)				0.005
Junior high school or less	1974 (49.2)	1483 (75.1)	491 (24.9)	
High school	1085 (27.0)	847 (78.1)	238 (21.9)	
College or above	715 (17.8)	543 (75.9)	172 (24.1)	
Unknown/Refuse to answer	239 (6.0)	161 (67.4)	78 (32.6)	
Maternal educational, n (%)				0.016
Junior high school or less	2350 (58.6)	1775 (75.5)	575 (24.5)	
High school	841 (21.0)	658 (78.2)	183 (21.8)	
College or above	576 (14.4)	433 (75.2)	143 (24.8)	
Unknown/Refuse to answer	246 (6.1)	168 (68.3)	78 (31.7)	
Household income, n (%), yuan				0.211
<30,000	1065 (26.5)	781 (73.3)	284 (26.7)	
30,000–80,000	1172 (29.2)	888 (75.8)	284 (24.2)	
80,000–150,000	1106 (27.6)	839 (75.9)	267 (24.1)	
≥150,000	670 (16.7)	526 (78.5)	144 (21.5)	
Academic stress, n (%)				<0.001
None	213 (5.3)	169 (79.3)	44 (20.7)	
Low	187 (4.7)	141 (75.4)	46 (24.6)	
Somewhat low	285 (7.1)	232 (81.4)	53 (18.6)	
Moderate	2474 (61.6)	1889 (76.4)	585 (23.6)	
Somewhat high	532 (13.3)	397 (74.6)	135 (25.4)	
High	121 (3.0)	80 (66.1)	41 (33.9)	
Unknown/Refuse to answer	201 (5.0)	126 (62.7)	75 (37.3)	
Physical activity, n (%)				<0.001
No	2375 (59.2)	1749 (73.6)	626 (26.4)	
Yes	1638 (40.8)	1285 (78.4)	353 (21.6)	
Alcohol consumption, n (%)				0.056
No	3976 (99.1)	3011 (75.7)	965 (24.3)	
Yes	37 (0.9)	23 (62.2)	14 (37.8)	
SHS exposure, n (%)				0.021
No	3682 (91.8)	2801 (76.1)	881 (23.9)	
Yes	331 (8.2)	233 (70.4)	98 (29.6)	
Frequency of SHS exposure, n (%)				<0.001
Never (0)	439 (12.3)	400 (81.1)	93 (18.9)	
Very infrequent (1-3/month)	1125 (28.0)	885 (78.7)	240 (21.3)	
Infrequent (1-2/week)	915 (22.8)	687 (75.1)	228 (24.9)	
Moderate (3-4/week)	886 (22.1)	634 (71.6)	252 (28.4)	
Fairly frequent (5-6/week)	338 (8.4)	249 (73.7)	89 (26.3)	
Very frequent (≥1/day)	256 (6.4)	179 (69.9)	77 (30.1)	
Number of surrounding smokers, n(%)				<0.001
0	2359 (58.8)	1842 (78.1)	517 (21.9)	
1–4	1223 (30.5)	903 (73.8)	320 (26.2)	
5–9	215 (5.4)	151 (70.2)	64 (29.8)	
≥10	216 (5.4)	138 (63.9)	78 (36.1)	
Frequency of THS exposure, n(%)				<0.001
Never (0)	929 (23.1)	740 (79.7)	189 (20.3)	
Very infrequent (1-3/month)	1298 (32.3)	1026 (79.0)	272 (21.0)	
Infrequent (1-2/week)	787 (19.6)	563 (71.5)	224 (28.5)	
Moderate (3-4/week)	658 (16.4)	472 (71.7)	186 (28.3)	
Fairly frequent (5-6/week)	205 (5.1)	135 (65.9)	70 (34.1)	
Very frequent (≥1/day)	136 (3.4)	98 (72.1)	38 (27.9)	
BRIEF-A scales, mean (SD)				
BRI score	11.5 (12.5)	7.2 (9.8)	25.0 (10.3)	<0.001
MI score	15.9 (16.5)	10.2 (13.1)	33.4 (13.5)	<0.001
GEC score	27.4 (28.9)	17.4 (22.6)	58.4 (23.7)	<0.001
ADHD symptoms, n(%)				
No	3034 (75.6)			
Yes	979 (24.4)			

BMI, Body Mass Index; SHS, secondhand smoke; THS, thirdhand smoke; BRI, Behavioral Regulation Index; MI, Metacognitive Index; GEC, Global Executive Composite; ADHD, attention-deficit/hyperactivity disorder.

*P* value from t-test for continuous variable or chi-square test for categorical variable.

### 3.2 SHS/THS exposure and ADHD symptoms

The associations between SHS/THS exposure and ADHD symptoms are presented in **Tables 2** and **3**. In the fully adjusted model, compared with unexposed, participants ever exposed to SHS ≥15 min at least 1 day/week had higher odds of ADHD symptoms (OR, 1.31; 95% CI, 1.02–1.67). In addition, the frequency of SHS/THS exposure and the number of surrounding smokers were also significantly related to the ADHD symptoms. Compared to the participants who had never been exposed to SHS/THS, those with SHS exposure ≥1/day (OR, 1.80; 95% CI, 1.26–2.56)) and those with THS exposure 5–6/week (OR, 1.98; 95% CI, 1.42, 2.77) had significantly increased odds of ADHD symptoms. Meanwhile, the risk of ADHD symptoms was increased accompanying with the number of surrounding smoker (OR, 1.89; 95% CI, 1.40, 2.55).

**Table 2:** The association of secondhand smoke exposure and the ADHD symptoms.

SHS Exposure	Crude^a^		Adjusted^b^	
	ORs (95%CI)	*P* value	ORs (95%CI)	*P* value
SHS exposure				
No	ref	ref	ref	ref
Yes	1.39 (1.09, 1.77)	0.008	1.31 (1.02, 1.67)	0.034
Number of surrounding smokers				
0	ref	ref	ref	ref
1–4	1.25 (1.07, 1.47)	0.006	1.29 (1.09, 1.52)	0.002
5–9	1.57 (1.16, 2.13)	0.004	1.52 (1.12, 2.07)	0.008
≥10	1.96 (1.46, 2.62)	<0.001	1.89 (1.40, 2.55)	<0.001
Frequency of SHS exposure				
0	ref	ref	ref	ref
1–3/month	1.17 (0.89, 1.53)	0.252	1.20 (0.91, 1.57)	0.191
1–2/week	1.43 (1.09, 1.87)	0.010	1.46 (1.11, 1.92)	0.007
3–4/week	1.72 (1.32, 2.25)	<0.001	1.74 (1.32, 2.28)	<0.001
5–6/week	1.54 (1.10, 2.14)	0.011	1.50 (1.07, 2.10)	0.018
≥1/day	1.91 (1.35, 2.70)	<0.001	1.80 (1.26, 2.56)	0.001
*P* for trend	<0.001		<0.001	

SHS, secondhand smoke; ADHD, attention-deficit/hyperactivity disorder; OR, odds ratio; CI, confidence interval.

These results were analyzed using logistic regression models.

^a^Unadjusted models. ^b^Adjusted for age, sex, race, BMI, parental education, home address, physical activity, academic stress, and alcohol consumption.

**Table 3:** The association of thirdhand smoke exposure and the ADHD symptoms.

Frequency of THS exposure	Crude^a^		Adjusted^b^	
	ORs (95%CI)	*P* value	ORs (95%CI)	*P* value
0	ref	ref	ref	ref
1–3/month	1.04 (0.85, 1.28)	0.704	1.06 (0.86, 1.30)	0.614
1–2/week	1.55 (1.24, 1.94)	<0.001	1.55 (1.24, 1.94)	<0.001
3–4/week	1.56 (1.24, 1.97)	<0.001	1.54 (1.22, 1.95)	<0.001
5–6/week	2.08 (1.50, 2.88)	<0.001	1.98 (1.42, 2.77)	<0.001
≥1/day	1.60 (1.07, 2.39)	0.022	1.44 (0.96, 2.17)	0.079
*P* for trend	<0.001		<0.001	

THS, thirdhand smoke; ADHD, attention-deficit/hyperactivity disorder; OR, odds ratio; CI, confidence interval.

These results were analyzed using logistic regression models.

^a^Unadjusted models. ^b^Adjusted for age, sex, race, BMI, parental education, home address, physical activity, academic stress, and alcohol consumption.

### 3.3 SHS/THS exposure and executive function

**Tables 4**-**6** present the associations between SHS/THS exposure and executive function. Higher scores, indicating poorer executive function in behavior regulation, metacognition, and global executive functioning were observed in participants with SHS/THS exposure. In the fully adjusted model (**Table 5**), SHS exposure ≥15 min at least 1 day/week was significantly associated with higher BRI score (β, 2.34; 95% CI, 0.95–3.73), higher MI score (β, 3.12; 95% CI, 1.29–4.95), and higher GEC score (β, 5.46; 95% CI, 2.26–8.66). In addition, the greater number of surrounding smoker, the higher score of BRI (β, 3.84; 95% CI, 2.11–5.57), MI (β, 4.85; 95% CI, 2.57–7.13), and GEC (β, 8,69; 95% CI, 4.72–12.67). Furthermore, higher SHS/THS exposure frequency was a significant predictor of impaired executive function. As shown in **Tables 5** and **6**, SHS exposure ≥1/day was significantly associated with higher BRI score (β, 4.50; 95% CI, 2.63–6.37), higher MI score (β, 6.10; 95% CI, 3.63–8.56), and higher GEC score (β, 10.60; 95% CI, 6.29–14.90). Similarly, THS exposure ≥1/day was significantly associated with higher BRI score (β, 3.05; 95% CI, 0.82–5.28), higher MI score (β, 4.10; 95% CI, 1.16–7.04), and higher GEC score (β, 7.15; 95% CI, 2.02–12.28).

**Table 4:** The association of secondhand smoke exposure and the executive function scores in crude model.

SHS exposure	BRI score		MI score		GEC score	
	β (95%CI)	*P value*	β (95%CI)	*P value*	β (95%CI)	*P value*
SHS exposure						
No	ref	ref	ref	ref	ref	ref
Yes	2.62 (1.24, 4.01)	<0.001	3.50 (1.67, 5.33)	<0.001	6.12 (2.93, 9.32)	<0.001
Number of surrounding smokers						
0	ref	ref	ref	ref	ref	ref
1–4	1.88 (1.02, 2.74)	<0.001	2.69 (1.56, 3.83)	<0.001	4.58 (2.60, 6.55)	<0.001
5–9	4.60 (2.87, 6.33)	<0.001	6.10 (3.82, 8.38)	<0.001	10.70 (6.72, 14.68)	<0.001
≥10	4.25 (2.52, 5.97)	<0.001	5.38 (3.10, 7.65)	<0.001	9.62 (5.65, 13.60)	<0.001
Frequency of SHS exposure						
0	ref	ref	ref	ref	ref	ref
1–3/month	2.03 (0.71, 3.35)	0.003	2.76 (1.02, 4.50)	0.002	4.79 (1.75, 7.82)	0.002
1–2/week	2.66 (1.30, 4.03)	<0.001	3.58 (1.78, 5.38)	<0.001	6.24 (3.10, 9.39)	<0.001
3–4/week	4.05 (2.68, 5.43)	<0.001	5.24 (3.43, 7.05)	<0.001	9.29 (6.13, 12.45)	<0.001
5–6/week	4.32 (2.60, 6.04)	<0.001	5.63 (3.36, 7.89)	<0.001	9.94 (5.98, 13.90)	<0.001
≥1/day	5.13 (3.26, 6.99)	<0.001	7.02 (4.56, 9.48)	<0.001	12.14 (7.85, 16.44)	<0.001
*P* for trend	<0.001		<0.001		<0.001	

SHS, secondhand smoke; BRI, Behavioral Regulation Index; MI, Metacognitive Index; GEC, Global Executive Composite; CI: confidence interval.

These results were analyzed using linear regression models.

**Table 5:** The association of secondhand smoke exposure and the executive function scores in adjusted model.

SHS exposure	BRI score		MI score		GEC score	
	β (95%CI)	*P value*	β (95%CI)	*P value*	β (95%CI)	*P value*
SHS exposure						
No	ref	ref	ref			ref
Yes	2.34 (0.95, 3.73)	0.001	3.12 (1.29, 4.95)	0.001	5.46 (2.26, 8.66)	0.001
Number of surrounding smokers						
0	ref	ref	ref			ref
1–4	1.82 (0.96, 2.68)	<0.001	2.61 (1.48, 3.74)	<0.001	4.43 (2.46, 6.41)	<0.001
5–9	4.23 (2.51, 5.96)	<0.001	5.56 (3.29, 7.84)	<0.001	9.80 (5.82, 13.77)	<0.001
≥10	3.84 (2.11, 5.57)	<0.001	4.85 (2.57, 7.13)	<0.001	8.69 (4.72, 12.67)	<0.001
Frequency of SHS exposure						
0	ref	ref	ref			ref
1–3/month	1.93 (0.61, 3.25)	0.004	2.62 (0.88, 4.36)	0.003	4.55 (1.51, 7.58)	0.003
1–2/week	2.54 (1.17, 3.90)	<0.001	3.40 (1.60, 5.20)	<0.001	5.93 (2.79, 9.08)	<0.001
3–4/week	3.74 (2.36, 5.11)	<0.001	4.77 (2.96, 6.58)	<0.001	8.50 (5.34, 11.67)	<0.001
5–6/week	3.85 (2.12, 5.57)	<0.001	5.01 (2.74, 7.28)	<0.001	8.86 (4.89, 12.83)	<0.001
≥1/day	4.50 (2.63, 6.37)	<0.001	6.10 (3.63, 8.56)	<0.001	10.60 (6.29, 14.90)	<0.001
*P* for trend	<0.001		<0.001		<0.001	

SHS, secondhand smoke; BRI, Behavioral Regulation Index; MI, Metacognitive Index; GEC, Global Executive Composite; CI: confidence interval.

These results were analyzed using linear regression models, adjusted for age, sex, race, BMI, parental education, home address, physical activity, academic stress, and alcohol consumption.

**Table 6:** The association of thirdhand smoke exposure and the executive function scores.

Frequency of THS exposure	BRI score		MI score		GEC score	
	β (95%CI)	*P value*	β (95%CI)	*P value*	β (95%CI)	*P value*
Crude^a^						
0	ref	ref	ref	ref	ref	ref
1–3/month	1.42 (0.38, 2.47)	0.008	1.86 (0.48, 3.24)	0.008	3.28 (0.87, 5.70)	0.008
1–2/week	3.24 (2.06, 4.42)	<0.001	4.19 (2.63, 5.75)	<0.001	7.43 (4.71, 10.15)	<0.001
3–4/week	4.01 (2.77, 5.25)	<0.001	4.97 (3.33, 6.60)	<0.001	8.98 (6.13, 11.83)	<0.001
5–6/week	5.45 (3.58, 7.32)	<0.001	7.21 (4.74, 9.68)	<0.001	12.66 (8.35, 16.96)	<0.001
≥1/day	3.67 (1.45, 5.90)	0.001	4.98 (2.04, 7.92)	0.001	8.65 (3.53, 13.78)	0.001
*P* for trend	<0.001		<0.001		<0.001	
Adjusted^b^						
0	ref	ref	ref			ref
1–3/month	1.38 (0.33, 2.42)	0.010	1.79 (0.41, 3.16)	0.011	3.16 (0.76, 5.57)	0.01
1–2/week	3.11 (1.93, 4.29)	<0.001	3.98 (2.43, 5.53)	<0.001	7.09 (4.38, 9.81)	<0.001
3–4/week	3.75 (2.51, 4.99)	<0.001	4.61 (2.98, 6.24)	<0.001	8.36 (5.51, 11.21)	<0.001
5–6/week	5.02 (3.14, 6.89)	<0.001	6.62 (4.14, 9.09)	<0.001	11.64 (7.32, 15.95)	<0.001
≥1/day	3.05 (0.82, 5.28)	0.007	4.10 (1.16, 7.04)	0.006	7.15 (2.02, 12.28)	0.006
*P* for trend	<0.001		<0.001		<0.001	

THS, thirdhand smoke; BRI, Behavioral Regulation Index; MI, Metacognitive Index; GEC, Global Executive Composite; CI: confidence interval.

These results were analyzed using linear regression models.

^a^Unadjusted models. ^b^Adjusted for age, sex, race, BMI, parental education, home address, physical activity, academic stress, and alcohol consumption.

## 4 Discussion

In this cross-sectional study, we found that SHS and THS exposure were associated with higher risk of ADHD symptoms and poorer executive function at young adults, even after adjustment for important covariates. Whether ever exposed to SHS, or the frequency of SHS/THS exposure, or the number of surrounding smokers were significantly associated with the ADHD symptoms and the BRIEF-A scores (BRI, MI, and GEC). The risk of ADHD symptoms and the executive function scores were increased accompanying with the frequency of SHS/THS exposure and the number of surrounding smokers.

Despite a gradual decline in smoking rates over time, exposure to SHS continues to cause harm to nonsmokers, such as the increased disease burden of cardiovascular disease, cancer, respiratory conditions, type 2 diabetes (7). In recent years, the health impact of SHS on mental health and neurological function has attracted public concern (11). Lee S et al. found the significant association of SHS exposure and ADHD among 16434 children and adolescents (30). Some studies conducted in adolescents or school-aged children demonstrated SHS exposure was positively associated with higher risk of ADHD symptoms, depressive symptoms, loneliness, sleeplessness, and suicidal ideation (15, 33, 34). The adverse effect of SHS exposure on executive function was also reported. A prospective birth cohort study found that SHS exposure during pregnancy and childhood were associated with deficits in some domains of children’s executive function, especially task initiation and metacognition (16). Similarly, Rose-Jacobs R et al. found adverse effects of intrauterine tobacco exposure on executive function among high school students, which presented poor ability in metacognition and behavioral regulation (35).

Although growing evidence shows significant associations of SHS exposure with high risk of ADHD symptoms and poor executive function, most studies have focused on populations such as children or high school students, studies on SHS exposure and ADHD symptoms and executive function in early adulthood, a critical transitional phase of psychosocial development accompanied by unique stressors, are still limited. Meanwhile, some studies evaluated SHS exposure by a single question (e.g., weekly exposure days), without accounting for crucial factors such as the duration of exposure and the number of surrounding smokers, thus failing to fully capture the real SHS exposure. Therefore, the present study investigated the associations of SHS exposure with ADHD symptoms and executive function in non-smoking young adult, taking into account the exposure duration, the exposure frequency and the number of surrounding smokers. Findings showed that SHS exposure ≥15 min at least 1 day/week, the higher frequency of SHS exposure, and the greater number of surrounding smokers were significantly related to increased risk of ADHD symptoms and the decreased executive function, including behavioral regulation and metacognitive. These results make up the shortcomings of the previous research and provide evidence specifically for young adult. Also, the results are consistent with previous studies in population such as children or high school students.

In addition to SHS, THS has recently been recognized as a new threat to global public health. Some scholars have pointed out that the carcinogenic substance nitrosamines content in THS is 10 times higher than in SHS (36). Experimental studies found that THS exposure induced different effects such as damage in the liver and lungs, gastric function, or insulin resistance in mice (22, 37, 38). THS health effects include the induction of oxidative DNA damage in human cell lines, suggesting a probable influence on neurobehavioral systems (8). In recent years, studies focus on mental health effects related to THS exposure were limited, and most studies focused on children or pregnant women (2). Wang L et al. reported that THS exposure may be a risk factor for postpartum depression among pregnant women (39). However, research on the associations of THS with ADHD symptoms and executive function is scarce. As far as we know, this is the first epidemiology study to show the associations of THS exposure frequency with ADHD symptoms and executive function, indicating the toxicological profile of THS on nervous system, which was consistent with animal study (40). Despite the lack of standardized protocols for the THS assessment, our findings reveal a significant dose–response relationship (*P* for trend < 0.001) using exposure frequency. Further researches are warranted to verify these novel associations through multi-marker THS assessments, such as urinary cotinine and surface nicotine residues.

Although the mechanisms underlying the influence of SHS and THS on ADHD symptoms and executive function are yet to be elucidated, the plausible explanation might be the adverse effects of hazardous chemical substances released by SHS and THS, such as nicotine, heavy metals, and polyaromatic hydrocarbon (2). First, these substances have been shown to dysregulate neurotransmitters such as dopamine and norepinephrine, which are necessary for the regulation of attention, motivation, and inhibitory control (24). In addition, nicotine binds to nicotinic acetylcholine receptors (nAChRs) in the brain, which are densely expressed in regions like the prefrontal cortex, striatum, and hippocampus, areas central to executive function and reward processing (41). The activation of the nAChRs subsequently results in the activation of NADPH oxidase, leading to the generation of reactive oxygen species, which oxidize lipids, proteins, and DNA in neurons. In the prefrontal cortex and hippocampus, this leads to mitochondrial dysfunction and neuronal apoptosis, directly compromising cognitive function (42). Furthermore, hazardous chemical released by SHS and THS triggers the production of pro-inflammatory cytokines, which disrupts the blood-brain barrier, allowing more toxins to enter the brain and exacerbating neuronal damage (43). Future researches are warranted to explore the biological mechanisms underlying the adverse effects of SHS and THS.

Our findings have critical public health implications. The harm of SHS and THS on ADHD and executive function may be independent of demographic variables, lifestyles, parental factors, and academic stress. Therefore, policies aimed at reducing tobacco smoke exposure in early adulthood should extend beyond banning active smoking to include measures targeting SHS and THS, which are harmful to mental and physical health. In addition, many young adults are aware of the harms of SHS, but the detrimental impact of THS have not been fully understood (5). The present study indicated dose–response associations between THS exposure frequency and the risk of ADHD symptoms and the poor executive function. Health education campaigns should raise awareness about THS, extending smoke-free regulations beyond public indoor spaces to include multi-unit housing and dormitories where young adults spend substantial time. In a word, policies and technical measures should be performed by social and government to reduce the production and spread of SHS and THS, and colleges and vocational institutes should consider integrating reduction strategies on SHS and THS exposure into campus mental-health plans.

Several limitations should not be ignored. First, the self-reported SHS and THS exposure might introduce recall bias, further studies should incorporate objective biomarkers (e.g., urine cotinine, surface nicotine, indoor air monitoring) to validate exposure. Second, the self-reported ADHD and executive function tasks might underestimate subtle deficits detectable only with laboratory-based neuropsychological testing. Third, residual confounding by unmeasured variables, such as interpersonal relationships, concurrent substance use, family psychiatric history might influence the study findings. In addition, the lack of a specific assessment for pre-existing mental health conditions represents a significant methodological limitation. Although we adjusted for several socioeconomic and lifestyle factors associated with mental health, residual confounding might bias the observed associations. Future studies should incorporate standardized clinical assessments of mental health. Meanwhile, some proxy measures of air quality which might influence the SHS and THS exposure, such as proximity to industrial areas, ventilation characteristics of living environment, was not be estimated. Furthermore, the cross-sectional design could not establish a causal relationship between SHS/THS exposure and ADHD symptoms and executive function in young adults, and the observed association requires further verification by longitudinal studies. Finally, our participants recruiting from vocational-college may limit generalizability to more populations.

## 5 Conclusions

The present study showed that SHS and THS exposure was associated with the occurrence of ADHD symptoms and the impaired executive function. Associations found does not prove causality considering the design is cross-sectional, thus further investigations across more diverse populations are needed to validate these associations.

## Data Availability

The raw data supporting the conclusions of this article will be made available by the authors, without undue reservation.
